# Herceptin Conjugates Linked by EDC Boost Direct Tumor Cell Death via Programmed Tumor Cell Necrosis

**DOI:** 10.1371/journal.pone.0023270

**Published:** 2011-08-10

**Authors:** Jiemiao Hu, Xinli Liu, Dennis Hughes, Francisco J. Esteva, Bolin Liu, Joya Chandra, Shulin Li

**Affiliations:** 1 Department of Pediatric Research, The University of Texas MD Anderson Cancer Center, Houston, Texas, United States of America; 2 Department of Pharmaceutical Sciences, School of Pharmacy, Texas Tech University Health Sciences Center, Amarillo, Texas, United States of America; 3 Department of Breast Medical Oncology, The University of Texas MD Anderson Cancer Center, Houston, Texas, United States of America; 4 Department of Pathology, University of Colorado Denver School of Medicine, Aurora, Colorado, United States of America; Roswell Park Cancer Institute, United States of America

## Abstract

Tumor-targeted antibody therapy is one of the safest biological therapeutics for cancer patients, but it is often ineffective at inducing direct tumor cell death and is ineffective against resistant tumor cells. Currently, the antitumor efficacy of antibody therapy is primarily achieved by inducing indirect tumor cell death, such as antibody-dependent cell cytotoxicity. Our study reveals that Herceptin conjugates, if generated via the crosslinker EDC (1-ethyl-3-(3-dimethylaminopropyl) carbodiimide hydrochloride), are capable of engendering human epidermal growth factor receptor 2 (Her2) positive tumor cells death. Using a high-performance liquid chromatography (HPLC) system, three peaks with estimated molecular weights of antibody monomer, dimer, and trimer were isolated. Both Herceptin trimer and dimer separated by HPLC induced significant levels of necrotic tumor cell death, although the trimer was more effective than the dimer. Notably, the Herceptin trimer also induced Herceptin-resistant tumor cell death. Surprisingly different from the known cell death mechanism that often results from antibody treatment, the Herceptin trimer elicited effective and direct tumor cell death via a novel mechanism: programmed cell necrosis. In Her2-positive cells, inhibition of necrosis pathways significantly reversed Herceptin trimer-induced cell death. In summary, the Herceptin trimer reported herein harbors great potential for overcoming tumor cell resistance to Herceptin treatment.

## Introduction

Human epidermal growth factor receptor 2 (Her2) is overexpressed in many types of cancers. [1], [2]. Herceptin, also known as Trastuzumab, is a humanized recombinant monoclonal antibody that binds to the extracellular domain of Her2 and is the first Her2 antibody approved by the US Food and Drug Administration for treating cancers in humans. Antibody therapy provides excellent tumor specificity; however, the clinical response to Herceptin therapy has not been very strong, with only 12–34% tumor remission noted over 9 months in metastatic breast cancer patients in early clinical trials [3]. Moreover, both primary resistance and acquired resistance to Herceptin were observed, thus limiting broad application of this safe therapy [4]. Further improvement of Herceptin's therapeutic effect is needed.

Unlike chemotherapy, Herceptin does not directly cause tumor cell death. Like many other targeted antibodies, Herceptin induces Her2-positive tumor cell death via ADCC [5], [6], [7]. In the current study, we sought to test whether Herceptin conjugate promotes induction of direct tumor cell death and whether such effect may also overcome tumor resistance to antibody treatment.

We discovered that, regardless of tumor cell resistance to wild-type Herceptin, the Herceptin conjugate generated using EDC but not the crosslinker SMCC (succinimidyl-4-(N-maleimidomethyl)cyclohexane-1-carboxylate)as found by others [8], boosted direct tumor cell death via inducing programmed tumor cell necrosis. The Herceptin trimer conjugate was more effective than the Herceptin dimer conjugate in inducing Her2-positive tumor cell death. The Herceptin conjugate did not cause death of any Her2-negative or underexpressing tumor cells, illustrating this conjugate's specificity and potential safety as a therapeutic agent. To our knowledge, our report is the first to reveal the capability of a tumor-targeted antibody to simultaneously induce programmed necrotic tumor cell death (PNCD) and overcome the resistance of tumor cells to antibody treatment.

## Results

### Generation of Herceptin conjugates

**Figure 1:**
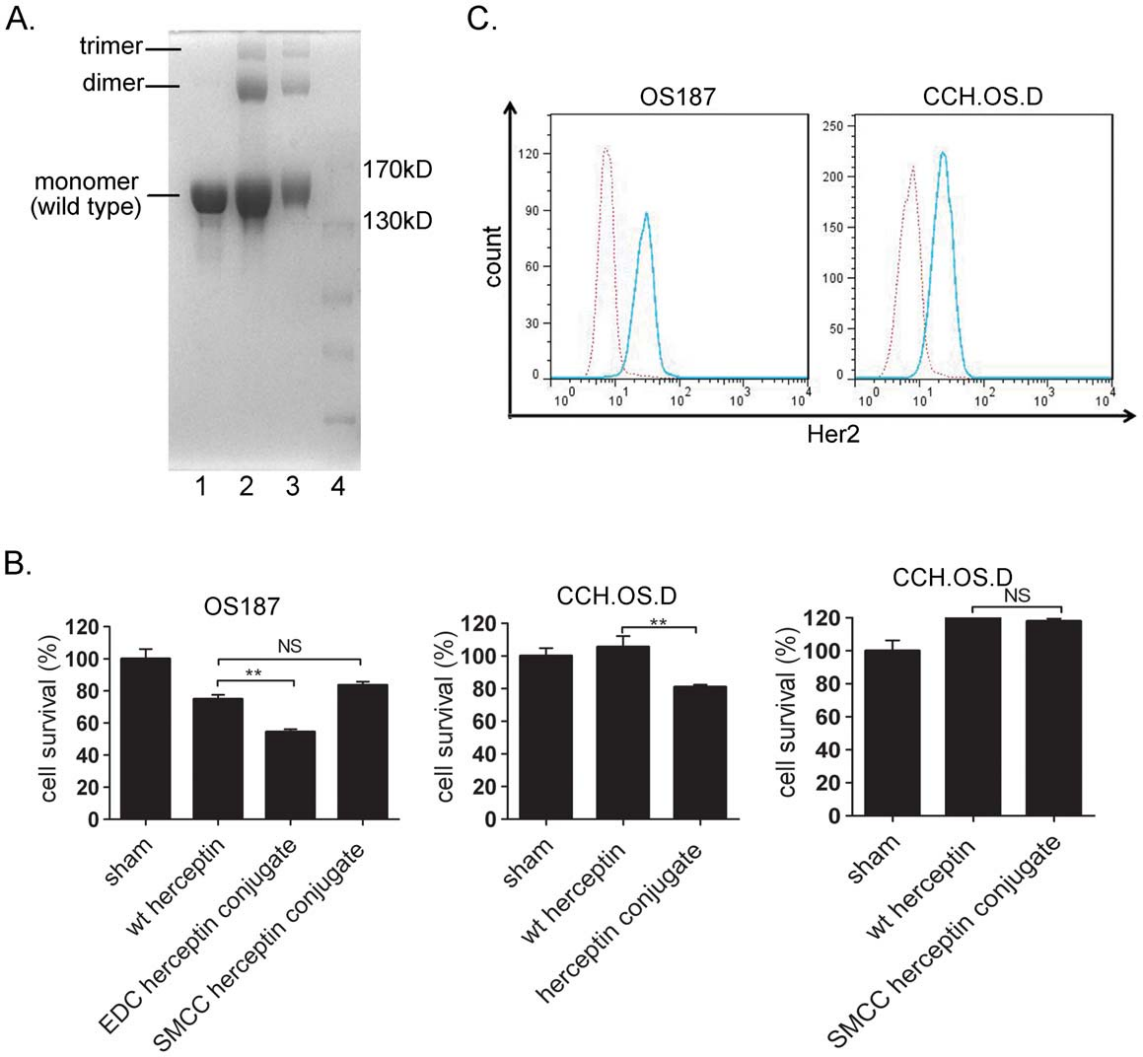
Suppression of Her2-positive tumor cell survival by EDC-Herceptin conjugate. (**a**) Gel analysis of the Herceptin conjugate. Analysis of Herceptin and Herceptin conjugates at 6% SDS-PAGE without reducing reagent DTT. Lane 1, wild-type Herceptin; lane 2, EDC-Herceptin conjugate; lane 3, SMCC-Herceptin conjugate; lane 4, molecular weight marker. (**b**) Reduction of Her2-positive tumor cell survival by EDC-Herceptin conjugate but not by SMCC-Herceptin conjugate. OS187 and CCH.OS.D cells were treated with the indicated molecules at 20 µg/mL for 72 h. MTT assay was used to determine the cell survival rate. (**c**) Her2 high expression cell lines OS187 and CCH.OS.D. Cells were stained with isotype control or PE-Her2 antibody. Her2 expression level was determined by using flow cytometry. Red dotted line, isotype control; blue line, Her2. *, *P*<0.05; **, *P*<0.01.

The anti-Her2 antibody, Herceptin, has proven effective in blocking the Her2 downstream signaling pathway [9], [10], [11] and in sensitizing Her2-expressing tumor cells to other treatments [12], [13]; however, there is no evidence that Herceptin alone induces potent tumor cell death. The hypothetical basis of this study tested whether the oligomerization of Herceptin immunoglobulin G (IgG) causes direct and effective tumor cell death. The rationale for generating this hypothesis preceded from the fact that immunoglobulin M (IgM), an immunoglobulin pentamer, causes tumor cell apoptosis [14], [15], [16]. To achieve Herceptin oligomers instead of a homodimers, we used a small crosslinker molecule, EDC, and deviated from the instructed time (2 h) and temperature (37°C) of the manufacturer when we generated the conjugates; we used a longer incubation time (4–6 h) at a lower temperature (room temperature). The conjugates contained three components with estimated molecular weights (MWs) of 148 kDa, 296 kDa, and 450 KDa on sodium dodecyl sulfate-polyacrylamide gel electrophoresis (SDS-PAGE) in the absence of the reducing agent dithiothreitol (DTT; Figure 1a, lane 2), suggesting that these proteins appeared in the conjugate may be an unconjugated wild-type antibody, a homodimer, and a homotrimer, respectively. Using another crosslinker, SMCC, we were able to generate the similar Herceptin oligomers as above by following the manufacturer's instruction (Figure 1a, lane 3).

### Suppression of tumor cell survival by the Herceptin conjugate treatment

To evaluate the role of conjugated Herceptin via two independent linkers, both wild-type Herceptin and Herceptin conjugates (20 µg/mL) were incubated with two independent Her2-positive cell lines, colon cancer line OS187 and osteosarcoma line CCH.OS.D (Figure 1c). Interestingly, the EDC-Herceptin conjugates greatly inhibited survival of both types of tumor cells (Figure 1b), whereas the SMCC conjugates failed to so inhibit tumor cell survival even though the conjugation pattern was the same on SDS-PAGE (Figure 1a). This observation led us to use the EDC linker for the rest of our experiments described in this study.

**Figure 2:**
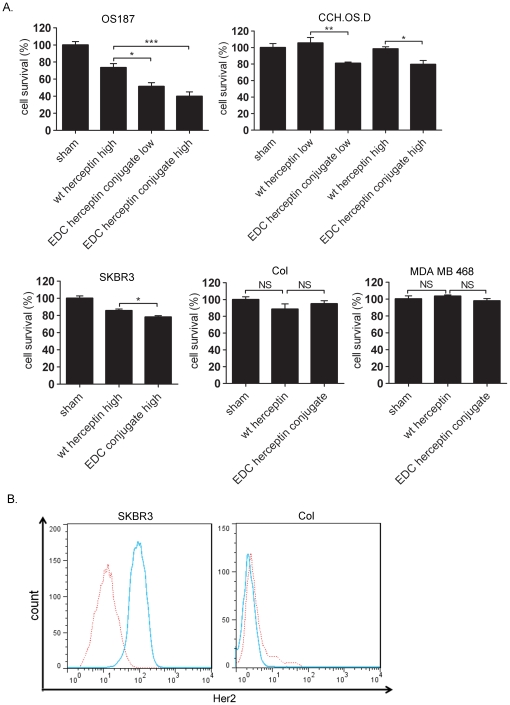
Treatment of Her2-positive or Her2-negative tumor cells by EDC-Herceptin conjugate. (**a**) Comparison of cell survival rates between wild-type Herceptin and EDC-Herceptin conjugate treatments in Her2-positive (OS187, CCH.OS.D, and SKBR3) and Her2-negative (Col and MDA-MB-468) cell lines. MTT assay was used after cells were treated with the described antibody at low concentration (20 µg/mL) or high concentration (80 µg/mL) for 72 h. (**b**) Her2 expression analysis in tested cell lines using flow cytometry as described in Figure 1. *, *P*<0.05; **, *P*<0.01; ***, *P*<0.001.

The reduction of tumor cell survival by the EDC-Herceptin conjugate was Her2-expression specific because no reduction of cell viability was observed in the two independent Her2-underexpressing cell lines, MDA-MB-468 [17] and Col (Figures 2a and b). In agreement with this observation, the inhibition of tumor cell survival by the EDC conjugate was dose dependent. We noticed that Her2-positive cell lines responded differently to treatment with the EDC-Herceptin conjugate. OS187 and CCH.OS.D cell lines, which were more sensitive, and we found that a low dose (20 µg/mL) treatment could reduce cell survival (Figure 2a). However, SKBR3 cells were less sensitive, and a high concentration (80 µg/mL) treatment had significant effect (Figure 2a).

### Treatment of Herceptin-resistant tumor cells using EDC Herceptin conjugate

**Figure 3:**
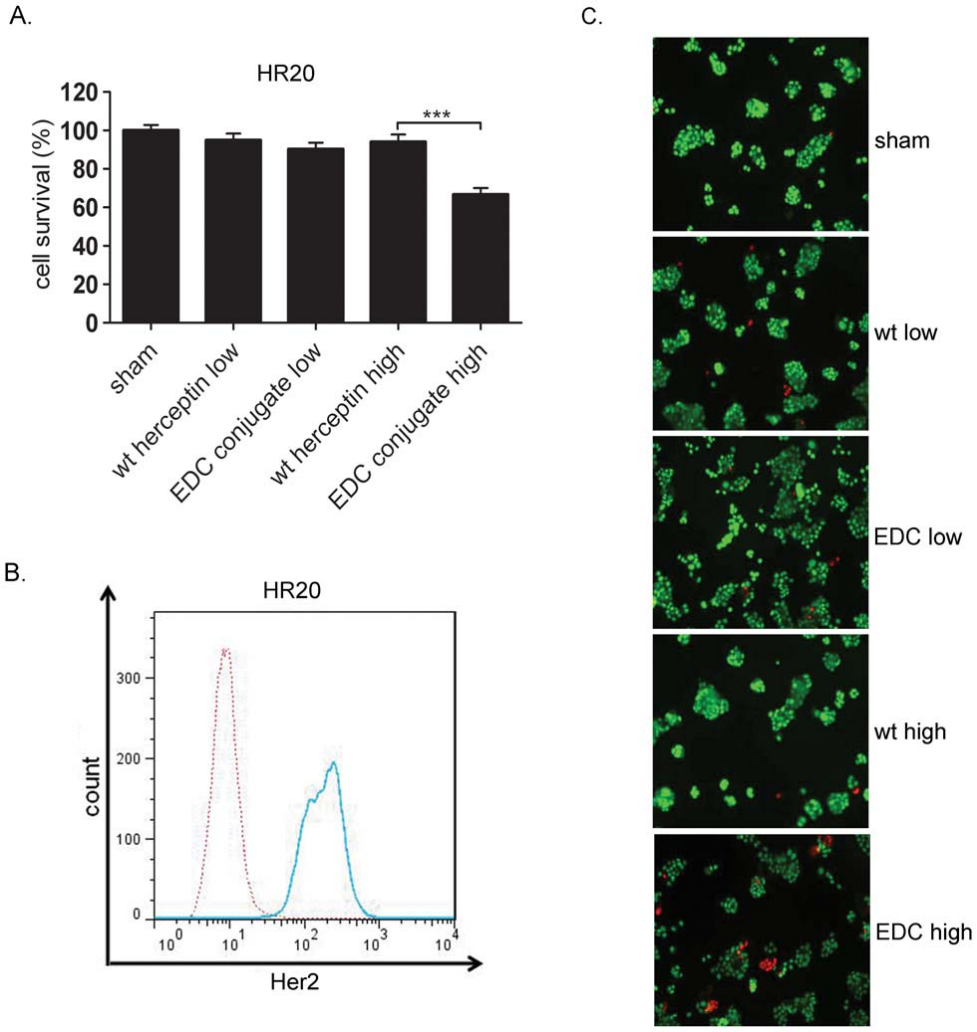
Induction of Herceptin-resistant tumor cell death by EDC-Herceptin conjugate. Herceptin-resistant tumor cells HR20 were treated with wild-type Herceptin or EDC conjugate at low concentration (20 µg/mL) or high concentration (80 µg/mL) for 72 h, and cell survival rates were analyzed using MTT assay. (**a**) Cell survival rate at the end of the described treatment. (**b**) Her2 expression level analyzed by flow cytometry as described in Figure 1. (**c**) Detection of cell death frequency following different treatments. Cells were treated with sham, wt low (wild-type Herceptin 20 µg/mL), EDC low (EDC-Herceptin conjugate 20 µg/mL), wt high (wild-type Herceptin 80 µg/mL), or EDC high (EDC-Herceptin conjugate 80 µg/mL). Live and dead cells were analyzed using a live/dead cell staining kit (see detail in Methods). Green fluorescence stained cells, live cells; Red florescence stained cells, dead and dying cells. ***, *P*<0.001.

Acquired resistance is one common mechanism by which tumor cells survive treatment and cause disease recurrence. For example, the majority of patients whose tumors overexpressed Her2 achieved an initial response to Herceptin but developed resistance within 1 year [18]. Bolin Liu's group [19] has developed a method to program tumor cells to acquire Herceptin resistance by continuously culturing BT474 cells with a gradually increasing concentration of Herceptin for 4 months. BT474-HR20 (HR20) cells have been defined as a Herceptin-resistant cell line and grow well in the presence of 20 µg/mL Herceptin [19]. In treating parental tumor cells, we found that treatment of the Her2-overexpressing (Figure 3b) but Herceptin-resistant tumor cell line HR20 with a high concentration of EDC-Herceptin conjugate suppressed tumor cell viability, whereas treatment with wild-type Herceptin at the same concentration failed to affect HR20 cell growth (Figure 3a). As determined with a fluorescence live/dead cell staining kit, more dead cells were detected in samples treated with the Herceptin conjugate than were detected in samples treated with the wild-type Herceptin (Figure 3c).

### Induction of tumor cell death but inhibition of tumor cell proliferation by EDC-Herceptin conjugate

**Figure 4:**
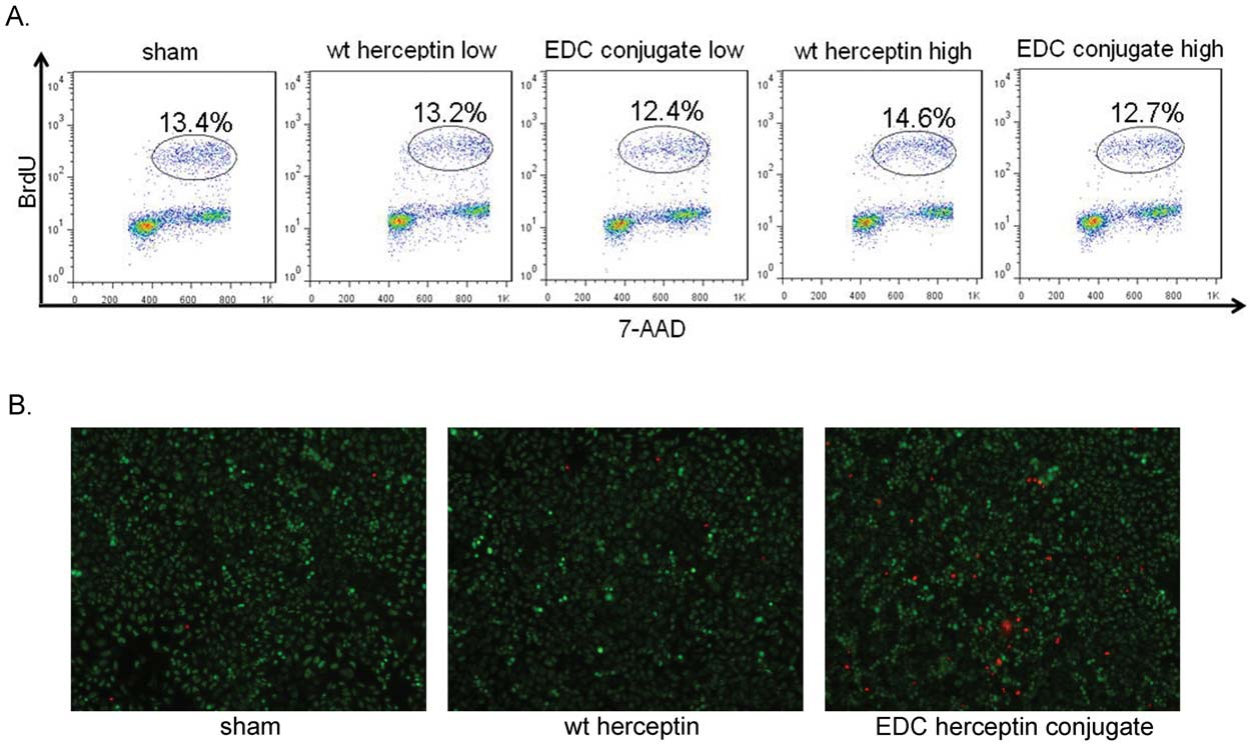
Induction of tumor cell death by EDC-Herceptin conjugate treatment. (**a**) BrdU incorporation rate following different treatments. Cells were treated for 72 h as described in Figure 2, followed by BrdU flow assay using flow cytometry. (**b**) Live and dead cell analysis on OS187 cells after treatment, as described in Figure 3.

To determine whether the reduction in cell survival was due to reduced cell proliferation or to increased cell death, a BrdU flow cytometric assay was performed as a marker of cell proliferation, and a cell death staining kit was used to distinguish the dead cells from the live cells. Cell proliferation was not greatly different after EDC-Herceptin conjugate treatment (Figure 4a). As we observed in HR20 cells, the EDC-Herceptin conjugate induced a great amount of cell death in OS187 cells (Figure 4b). This led us to conclude that the decreased cell survival rate after EDC-Herceptin conjugate treatment was due to the induction of cell death rather than to the inhibition of cell proliferation.

### Induction of necrosis-mediated tumor cell death and induction of late-stage apoptosis account for cell death induced by the EDC-Herceptin conjugates

Our results clearly indicated that the EDC-Herceptin conjugate significantly (P<0.05) induced Her2-positive tumor cell death, but the mechanism was unknown. Apoptosis is the most common cell death mechanism, and the origins of Herceptin conjugate-induced cell death could be attributed to the induction of apoptosis. To elucidate the mechanism of the cell death induction by the EDC-Herceptin conjugate, we chose an EDC-Herceptin conjugate treatment sensitive cell line (OS187) and a less sensitive cell line (SKBR3) for the following study.

**Figure 5:**
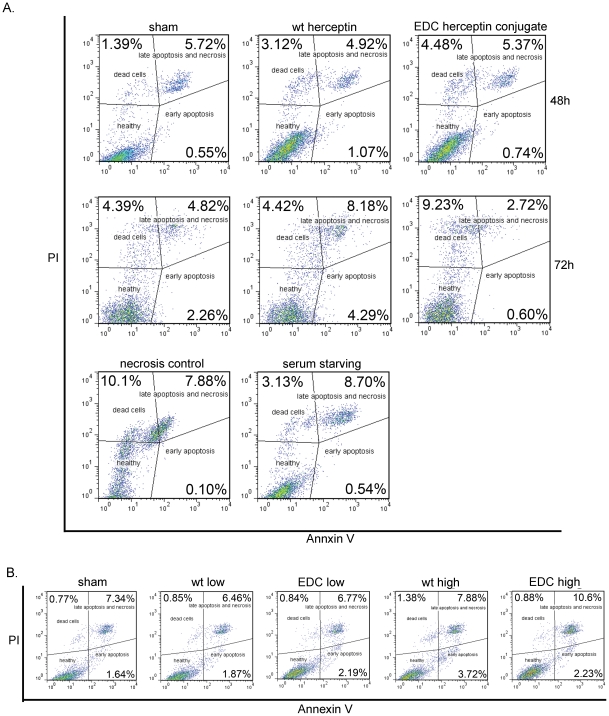
Induction of late-stage apoptosis and necrosis by EDC-Herceptin conjugate treatment. (**a**) OS187 cells were treated with sham, wt Herceptin (wild-type Herceptin), or EDC conjugate (EDC-Herceptin conjugate) for 48 or 72 h and then stained with annexin v and PI to determine the necrosis and apoptosis under flow cytometry. Necrosis control, freeze cells in −80°C for 5 min, and then thaw cells at 37°C. Serum starving, serum starving overnight as apoptosis control. (**b**) SKBR3 cells were treated as described in Figure 3, followed by PI, annexin v staining, and flow cytometry analysis.

To determine whether the apoptotic activity truly and solely accounted for the Herceptin conjugate-mediated tumor cell death, we used flow cytometry to compare the difference in cell death (both necrosis and apoptosis) between samples treated with wild-type Herceptin and samples treated with Herceptin conjugates. To detect the percentage of apoptotic cells and necrotic cells in the different antibody treatments, the treated cells were stained with fluorescein isothiocyanate (FITC)-annexin V and propidium iodide (PI) [20]. The cell population with both annexin V and PI negative represented viable cells. Cells stained with both annexin V and PI positive represented late apoptotic cells. The cell population with annexin V positive but PI negative represented early stage apoptotic cells. The cell population with PI positive but annexin V negative represented dead cells. In the OS187 cells, we discovered 48 h after treatment, compared with sham and wild-type Herceptin, the EDC conjugate induced a slight increase in the dead cell population (Figure 5a). 72 h after treatment, wild-type Herceptin-treated cells increased early and late stage apoptosis, while the EDC-Herceptin conjugate induced more than two times the number of dead cells compared with the sham and wild-type Herceptin (Figure 5a). In our necrosis control, we froze OS187 cells at −80°C for 5 min and then thawed the cells at 37°C. The severe freeze-thaw process devastated most cells so that cells tended to become necrotic. When we performed a PI-annexin V assay, we found EDC-Herceptin conjugate-treated cells had a trend similar to that of the necrosis control cells, suggesting that induction of necrosis might account for the EDC-Herceptin conjugate's effect. The SKBR3 cells were less sensitive to the EDC-Herceptin conjugate treatment. This result showed that, at a low dose (20 µg/mL), neither the wild-type Herceptin nor the EDC conjugate had much effect. However, high doses of the EDC-Herceptin conjugate greatly boosted the late apoptosis and necrosis cell population without any increase in early stage apoptosis (Figure 5b). Such results suggested that late apoptosis and necrosis might account for the EDC-Herceptin conjugate's effect on both cells lines. The difference of PI-annexin V staining between the two cell lines also explained the sensitivity difference of these two cells lines to EDC conjugate treatment. All the flow cytometry experiments were repeated at least three times with the same or similar results.

### Induction of apoptotic enzyme activities by EDC-Herceptin conjugates

**Figure 6:**
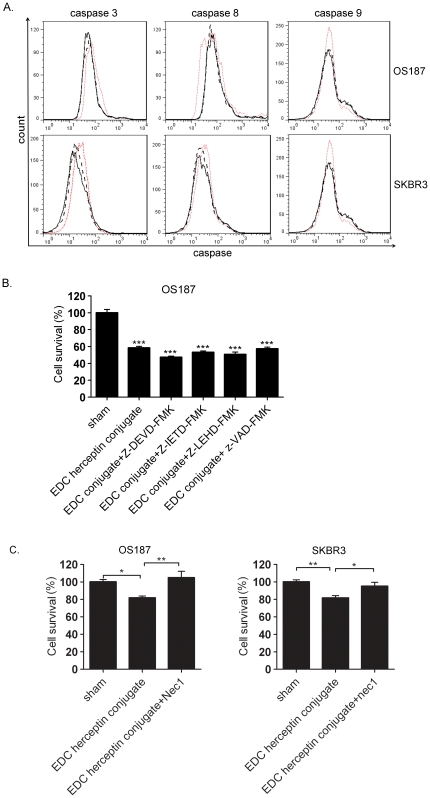
Analysis of cell death mechanism. (**a**) Active caspase analysis. OS187 and SKBR3 cells were treated as described in Figure 2. Caspase-3, -8, and -9 activity were tested by CaspGLOW fluorescein active caspase staining kits. Red dot line, sham; black dash line, wild-type Herceptin (80 µg/mL); black solid line, EDC-Herceptin conjugate (80 µg/mL). (**b**). Effect of caspase inhibitors on EDC conjugate-induced cell death. Z-DEVD-FMK, caspase 3 inhibitor. Z-IETD-FMK, caspase 8 inhibitor. Z-LEHD-FMK caspase 9 inhibitor. Z-VAD-FMK, caspase inhibitor. OS187 cells were treated with sham, EDC Herceptin conjugate 20 µg/mL or EDC Herceptin conjugate 20 µg/mL plus different caspase inhibitors at 30 µmol/L. (**c**) Effect of necrosis inhibitors on EDC-Herceptin conjugate-induced cell death. Necrostatin-1 (Nec-1), programmed cell necrosis inhibitor. OS187/SKBR3 cells were treated with sham, EDC-Herceptin conjugate 20/80 µg/mL, or EDC-Herceptin conjugate 20/80 µg/mL plus Nec-1 at 30 µmol/L. N = 5. *, *P*<0.05; **, *P*<0.01.

Our results indicated that apoptosis might contribute to the EDC conjugate-mediated Her2-positive tumor cell death. Apoptosis involves two major activation pathways [21], [22]: (1) internal via mitochondrial transmembrane stress and (2) external via death ligand binding activity. The former activates initiator caspase-9, and the latter activates caspase-8. Both caspase-9 and caspase-8 can activate apoptotic executor caspase-3. To determine the caspase pathway that could contribute to the EDC conjugate-mediated tumor cell death, the activated caspases 3, 8, and 9 were analyzed in both OS187 and SKBR3 cells after treatment. However, no difference was detected among sham, wild-type Herceptin, and EDC conjugate cells in both cell lines (Figure 6a). This flow cytometry result indicated that induction of cell apoptosis was not the main mechanism of EDC-Herceptin conjugate-mediated cell death. We confirmed this result by treating OS187 cells with EDC Hercpetin conjugate plus caspase 3 inhibitor, casepase 8 inhibitor, caspase 9 inhibitor or caspases inhibitor. Neither of the caspase inhibitors could reverse EDC Herceptin conjugate induced tumor cell death (Figure 6b), which suggested apoptosis didn't cause such cell death.

### Induction of RIP receptor-interacting protein-dependent PNCD by Herceptin conjugate treatment

Late apoptotic cells were positively stained for both apoptotic and necrosis markers. It would be interesting to know whether necrosis contributes to the EDC-Herceptin conjugate-induced cell death. Necrotic cell death occurs in two ways: (1) acute unprogrammed necrotic death and (2) PNCD. We were solely interested in detecting the PNCD because unprogrammed necrotic cell death typically causes an indiscriminate and massive cell death in an acute time period, which was not observed in our treatment. Our EDC-Herceptin conjugate induced solely Her2-specific tumor cell death. To test which cell death mechanism accounted for the EDC-Herceptin conjugate-induced cell death, a necrosis inhibitor was used to definitively determine that necrosis accounted for the Herceptin conjugate-mediated cell death. Necrostatin-1 (Nec-1), a potent and specific inhibitor for PNCD, was used because this inhibitor perturbs the key PNCD-regulator protein, called the receptor-interacting protein (RIP) [23]. The result confirmed our hypothesis because Nec-1 treatment reversed the cell survival reduction by Herceptin conjugate treatment as determined by 3-(4,5-dimethylthiazol-2-yl)-2,5-diphenyltetrazolium bromide (MTT) assay (Figure 6c), which suggested that necrosis was the crucial mechanism in EDC-Herceptin conjugate-induced Her2-positive tumor cell death.

### The critical component among the Herceptin conjugates for inducing direct tumor cell death

**Figure 7:**
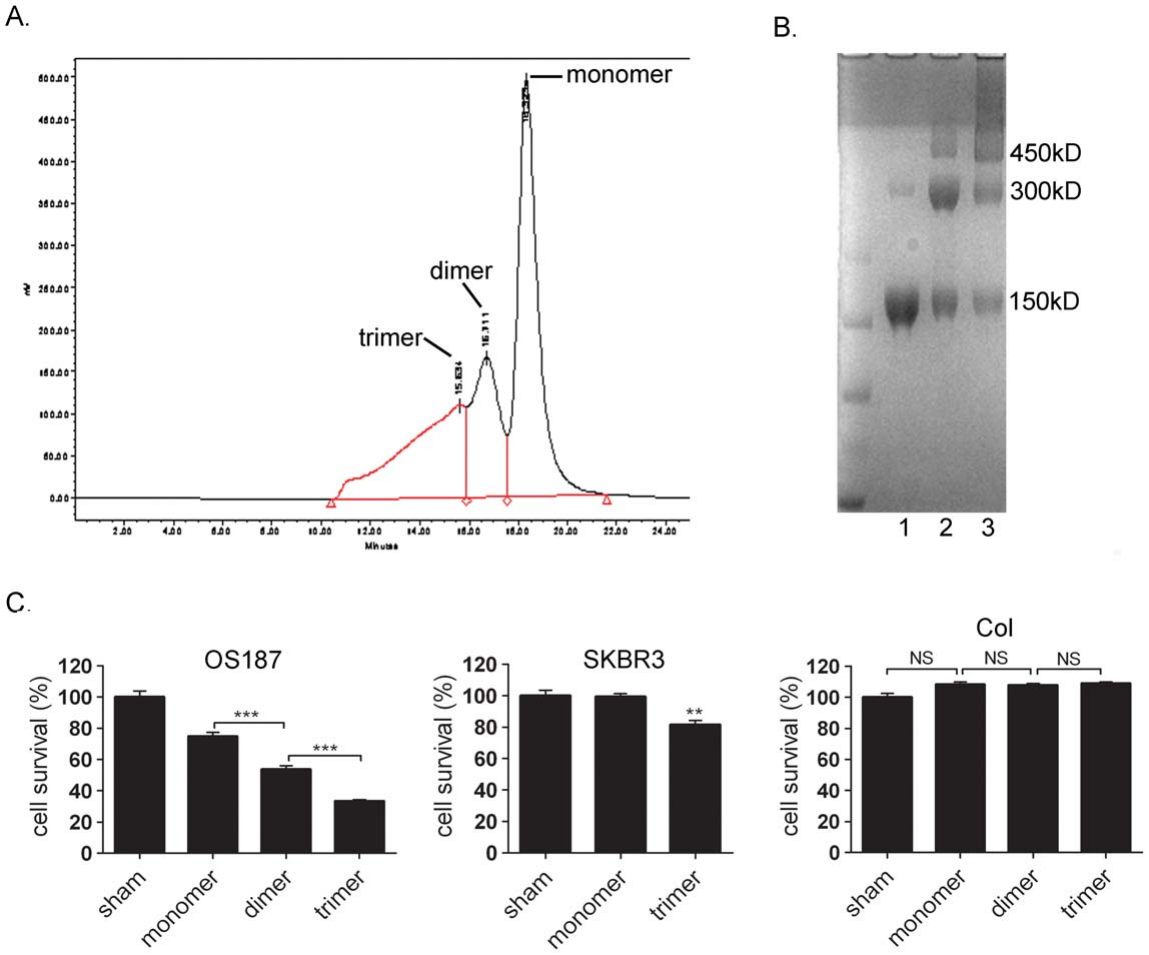
Identification of the primary conjugate fraction for triggering a significant amount of tumor cell death. (**a**) HPLC separation profile of the EDC-Herceptin conjugates. The dimer and trimer were estimated from the MWs of running wild-type Herceptin and the standard proteins. (**b**) Coomassie blue staining of separated EDC-Herceptin conjugate. 1, monomer; 2, dimer; 3, trimer. (**c**) Cell survival comparison following the treatments with Herceptin monomer, dimer, and trimer of the EDC-linked Herceptin (20 µg/mL). MTT assay was used. *, *P*<0.05; **, *P*<0.01; ***, *P*<0.001.

The EDC-Herceptin conjugates contained three different proteins: wild-type antibody (referred to as monomer), the fusion of two wild-type antibody (referred to as dimer), and the fusion of three antibody molecules (referred to as trimer) (Figure 1). The question was which form of the conjugate played the most significant role in inducing cell death. To this end, the mixture of the Herceptin conjugates was separated using a high-performance liquid chromatography (HPLC) system, and major peaks were found at the estimated retention times of 15, 16, and 17 min (Figure 7a). Based on the retention time of standard proteins with different MWs and wild-type antibodies run on the same HPLC separation system, it was determined that the fractions collected from the shorter retention time (14–15 min) contained a conjugate with an estimated MW of 450 kDa, which was the trimer antibody; fractions collected from the longer retention time (16 min) contained a conjugate with an estimated MW of 300 kDa, which was the dimer; and the fractions collected from the longest retention time of 17 min contained the monomer with an estimated MW of 150 kDa. Coomassie blue staining of the separated EDC-Herceptin conjugates verified the three conjugate components (Figure 7b). We noticed that these three components could not be completely isolated by HPLC. However, the key component (monomer, dimer, or trimer) was at least 50% of the total amount of proteins in each purified fraction, which was not perfect but was good enough for us to determine the effect of each individual protein. Treatment of OS187 and SKBR3 tumor cells with either purified Herceptin trimer or dimer, but especially the trimer, significantly increased tumor cell death compared with the wild-type antibody (Figure 7c). The EDC-Herceptin conjugated trimer was proved to be the most effective component in cell death induction. This dimer- or trimer-induced cell death was Her2-expression specific because no inhibition of cell viability was observed in the Her2-negative Col cell line. These results clearly suggested that the Herceptin trimer was the primary driving force causing PNCD.

## Discussion

Our results showed that only EDC-Herceptin conjugates promoted direct tumor cell death, while the SMCC conjugates failed to affect Her2-positive cell viability, which is different from the previous report [8]. This discrepancy in the SMCC conjugate result between us and the previous report from Ghetie *et al.* (1997) is perhaps due to a difference in conjugate conditions. In Ghetie *et al.* study, the monoclonal Her2 antibody Her-50 instead of Herceptin and two crosslinkers, SMCC and N-succinimidyl S-acetylthioacetate (SATA), were used to generate the homodimer. The homodimer HER-50 was reported to increase cell death via an apoptotic mechanism [8]. In our study, a low temperature (room temperature) and longer incubation time were employed. Our EDC conjugate induced PNCD. It is also possible that the EDC conjugate is more effective than the SMCC conjugate because the former generates a bond between the N-terminal from one antibody molecule and the C-terminal of another antibody molecule to form the dimer or trimer. As a result of this nature, the dimer or trimer is relatively relaxed by the EDC linker as compared with the SMCC linker, which yields thioether bonds by possibly using the cysteine in the hinge region of the antibody. The relaxed antibody has a longer extension to bind on different cells with the same conjugate, causing cell death.

Our study reveals for the first time that Herceptin, when forming a dimer or trimer via EDC, is capable of inducing PNCD, also called necroptosis. Necroptosis occurs naturally in embryonic development in addition to adult tissue homeostasis. It can be induced by tumor necrosis factor receptor (TNFR) activation, toll-like receptor (TLR) signaling, cytokines, and other factors. RIP kinase 1 serine/threonine kinase activity is required for death factor-mediated necroptosis [24] (Figure 5).

It is clear that PNCD is part of the mechanism by which the Herceptin dimer or trimer induces tumor cell death, but the details remain unclear. One possible model explaining the Herceptin trimer antibody-induced PNCD is that the Herceptin trimers bind multiple antigens in the same cell, changing the stability of the membrane. This model is supported by the fact that only IgM therapy induces direct tumor cell death and a therapeutic effect [14], [15], [16], [25]. Another possibility is that a single molecule binds to Her2 from different tumor cells, causing cell death. This theory is supported by the fact that IgM antibody against a B104 cell line illustrated a necrosis-like death [26].

To improve the potency of Herceptin therapy, it is critical to understand the mechanism responsible for antibody-induced tumor cell death and from where the resistance originates. Multiple mechanisms contribute to tumor cell resistance to Herceptin [19], [27], [28]. These resistant mechanisms were summarized into two categories by Liu's group [19]: (1) inhibition of antibody binding to the cognate receptors owing to truncation or the formation of homodimers and heterotrimers [19], [29], [30], [31] and (2) activation of Herceptin-resistant signaling pathways, such as activation of the phosphoinositide 3-kinase [32], and inactivation of Herceptin Herceptin-sensitive pathways, such as downregulation of p27kip1 and phosphatase and tensin homolog (PTEN) protein [19], [33]. Obviously, to overcome the tumor resistance to Herceptin, it is critical to overcome the binding resistance, the Herceptin-resistant signaling, or both. Huang *et al.* (2010) has found that Her2, human epidermal growth factor receptor 3 (Her3), and insulin-like growth factor 1 receptor (IGF-1R) formed a heterotrimeric complex in HR20 cells, which will likely cause conformational change of the Her2 receptor. Thus, EDC-Herceptin conjugates, particularly the Herceptin trimer, may have a better binding efficiency than wild-type Herceptin to the Her2 receptor in the heterotrimeric complex of Her2/Her3/IGF-1R. The published results also indicate that antibody modifications and antibodies combined with standard treatments could increase target receptor internalization, arresting tumor cell growth or enhancing tumor cell death [8], [23], [34], [35], [36], [37].

Our study illustrates an independent approach and mechanism to overcome the tumor resistance to antibody treatment and to induce necrotic cancer cell death. The induction of necroptosis has great potential to treat apoptosis-resistant cancer cells.

## Materials and Methods

### Ethics Statement

There are no human participants or animal work involved in this study.

### Cell culture and establishment of Herceptin resistant cells

The cancer cell lines SKBR3 and MDA-MB-468 were obtained from the American Type Culture Collection (ATCC, Rockville, MD, USA). The OS187 [38], CCH.OS.D, and Col [38] cells were provided by Dr Dennis PM Hughes's laboratory (The University of Texas MD Anderson Cancer Center, Houston, TX, USA). The CCH.OS.D is an osteosarcoma cell line derived from patients at the Children's Cancer Hospital at The University of Texas M.D. Anderson Cancer Center. Fingerprint analysis recently defined the OS187 colon cancer line a subline of HCT15 and the Col neuroblastoma line. HCT15 is available from the American Type Culture Collection (ATCC, Rockville, MD, USA). Herceptin-resistant cells HR20 were obtained as described by Dr Bolin Liu [19]. Cell lines were grown in Dulbecco's modified Eagle's medium (Mediatech, Inc., Manassas, VA, USA) supplemented with glutamine and heat-inactivated 10% fetal calf serum (FCS) and 10 U/mL penicillin and streptomycin.

### Herceptin conjugate generation

Herceptin was purchased from Genentech, Inc. (San Francisco, CA, USA). EDC (Pierce, Rockford, IL, USA) was the zero-length crosslinking agent used to couple carboxyl groups to primary amines. A quantity of 2 mg EDC was dissolved in 200 µL water and immediately mixed with 5 mg Herceptin, which was contained in the 400 µL reaction buffer of 0.1 M 2-(N-morpholino)-ethane sulfonic acid (pH 4.5) and incubated at room temperature for 4 h. Excess EDC and by-product were removed using amicon-50 KDa (Millipore, Billerica, MA, USA). SMCC-conjugated Herceptin was made as described in the manufacturer's SMCC instruction (Thermo Scientific, Rockford, IL, USA).

### SDS-PAGE analysis

Volumes containing 40 µg of protein were mixed with the same amount of 2× SDS loading buffer in the presence and absence of 10% DTT reducing reagent and added to wells of a 6% polyacrylamide gel, followed by the application of an electric field. The gel was then incubated with Coomassie Brilliant Blue R250 followed by destaining solution (10% acetic acid and 20% methanol). Images were captured with Quantity One version 4.4.1 software (Bio-Rad Laboratories, Hercules, CA, USA).

### Purification of Herceptin trimer complex using HPLC

The conjugation products were purified by a HPLC system using Waters Alliance 2690 with ultraviolet-absorbance detection at 280 nm. Trimers, dimers, and monomers were separated on the 300×7.8 mm Bio-Sil SEC-400-5 size exclusion column (Bio-Rad, Richmond, CA, USA) and were equilibrated in phosphate buffer (0.05 M monosodium phosphate [NaH_2_PO_4_], 0.05 M disodium hydrogen phosphate [Na_2_HPO_4_], and 0.15 M sodium chloride [NaCl], pH 6.8) at a flow rate of 0.5 mL/min. The protein fractions obtained were dialyzed overnight in distilled water at 4°C and lyophilized. A standard protein molecular mass profile was established using gel filtration standards (Bio-Rad) ranging from 1.3 to 670 KDa to identify the monomer, dimer, and trimer antibody peaks.

### Western blot analysis

One microgram of Herceptin and HPLC-separated Herceptin dimers or trimers from EDC- or SMCC-conjugates were mixed with 2× SDS-PAGE sample loading buffer, added to a 6% polyacrylamide gel, subjected to SDS-PAGE, and then transferred to a TransBlot Transfer Medium nitrocellulose membrane (Bio-Rad Laboratories). Immunoblotting of the membrane was performed with a 1∶100 dilution of polyclonal goat antihuman IgG containing secondary horseradish peroxidase. The peroxidase signal was generated by electrochemiluminescence using Western Lightning ECL (PerkinElmer, Inc., Walthman, MA, USA) and visualized with a Kodak Image Station 440CF, using the one-dimensional Image Analysis software version 3.6 (NEN Life Science Products, Inc., Boston, MA, USA).

### Live/dead cell assay

For the live/dead cell staining, cells were washed three times after treatments. The cell-permeable green fluorescent dye for staining the live cells and PI for staining the dead cells were each diluted in the staining buffer according to manufacturer's directions (Live/Dead Cell Staining Kit, BioVision, Inc., Mountain View, CA, USA). After 15 min incubation in 37°C, cells were observed under a fluorescence microscope (Olympus American, Inc., Center Valley, PA, USA).

### MTT assay

Cells (3×10^4^ per well, 5 replications for each treatment) with different treatments were incubated for 72 h. A quantity of 20 µL of MTT solution (5 mg/mL) was added to each well and incubated for 5 h prior to adding 200 µL of dimethyl sulfoxide (DMSO) to stop the reaction. The light absorbance was determined at 540 nm using a spectrometer (Packard Instrument, Inc., Downers Grove, IL, USA).

### BrdU flow assay

Cells (3×10^4^ per well, 5 replications for each treatment) with different treatments were incubated for 72 h. BrdU flow assay (APC BrdU flow kit, BD Biosciences, San Jose, CA, USA) was performed according to the manufacturer's directions.

### Caspase activity assays

CaspGLOW Green Caspase Staining Kit (BioVision, Inc.) was used to detect activated caspase-3, -8, or -9. The stained cells were analyzed using FACSCalibur flow cytometer (BD Biosciences). A total of 10,000 cells per sample were acquired, and data samples were analyzed using FlowJo software (version 7.6.1).

### Cell necrosis and apoptosis assays via flow cytometry

After treating the cells for 48 h, cells were gently trypsinized and washed once with serum-containing media. The cell pellets were washed with cold PBS (calcium [Ca^2+^] and magnesium [Mg^2+^] free) and binding buffer. The cell pellets were then resuspended in 500 µL binding buffer (10 mM 4-(2-hydroxyethyl)-1-piperazineethane sulfonic acid [HEPES], 140 mM NaCl, and 2.5 mM calcium chloride [CaCl_2_], pH 7.4) containing 5 µL FITC-annexin v and 5 µL PI in each sample, and cells were incubated on ice for 15 min. Samples were analyzed using flow cytometry.

### Statistical analysis

A one-way analysis of variance (ANOVA) test was used to determine statistical significance, followed by a post-hoc Tukey test to compare the means occurring between individual treatments. *P*<0.05 was considered statistically significant.

## References

[1] Press MF, Finn RS, Cameron D, Di Leo A, Geyer CE (2008). HER-2 gene amplification, HER-2 and epidermal growth factor receptor mRNA and protein expression, and lapatinib efficacy in women with metastatic breast cancer.. Clin Cancer Res.

[2] Press MF, Slamon DJ, Flom KJ, Park J, Zhou JY (2002). Evaluation of HER-2/neu gene amplification and overexpression: comparison of frequently used assay methods in a molecularly characterized cohort of breast cancer specimens.. J Clin Oncol.

[3] Goodwin PJ, Ligibel JA, Stambolic V (2009). Metformin in breast cancer: time for action.. J Clin Oncol.

[4] O'Brien NA, Browne BC, Chow L, Wang Y, Ginther C (2010). Activated phosphoinositide 3-kinase/AKT signaling confers resistance to trastuzumab but not lapatinib.. Mol Cancer Ther.

[5] Lewis GD, Figari I, Fendly B, Wong WL, Carter P (1993). Differential responses of human tumor cell lines to anti-p185HER2 monoclonal antibodies.. Cancer Immunol Immunother.

[6] Fendly BM, Kotts C, Vetterlein D, Lewis GD, Winget M (1990). The extracellular domain of HER2/neu is a potential immunogen for active specific immunotherapy of breast cancer.. J Biol Response Mod.

[7] Kurai J, Chikumi H, Hashimoto K, Yamaguchi K, Yamasaki A (2007). Antibody-dependent cellular cytotoxicity mediated by cetuximab against lung cancer cell lines.. Clin Cancer Res.

[8] Ghetie MA, Podar EM, Ilgen A, Gordon BE, Uhr JW (1997). Homodimerization of tumor-reactive monoclonal antibodies markedly increases their ability to induce growth arrest or apoptosis of tumor cells.. Proc Natl Acad Sci U S A.

[9] Baselga J, Albanell J, Molina MA, Arribas J (2001). Mechanism of action of trastuzumab and scientific update.. Semin Oncol.

[10] Sliwkowski MX, Lofgren JA, Lewis GD, Hotaling TE, Fendly BM (1999). Nonclinical studies addressing the mechanism of action of trastuzumab (Herceptin).. Semin Oncol.

[11] Nagata Y, Lan KH, Zhou X, Tan M, Esteva FJ (2004). PTEN activation contributes to tumor inhibition by trastuzumab, and loss of PTEN predicts trastuzumab resistance in patients.. Cancer Cell.

[12] Lee S, Yang W, Lan KH, Sellappan S, Klos K (2002). Enhanced sensitization to taxol-induced apoptosis by herceptin pretreatment in ErbB2-overexpressing breast cancer cells.. Cancer Res.

[13] Henson ES, Hu X, Gibson SB (2006). Herceptin sensitizes ErbB2-overexpressing cells to apoptosis by reducing antiapoptotic Mcl-1 expression.. Clin Cancer Res.

[14] Brandlein S, Lorenz J, Ruoff N, Hensel F, Beyer I (2002). Human monoclonal IgM antibodies with apoptotic activity isolated from cancer patients.. Hum Antibodies.

[15] Brandlein S, Pohle T, Ruoff N, Wozniak E, Muller-Hermelink HK (2003). Natural IgM antibodies and immunosurveillance mechanisms against epithelial cancer cells in humans.. Cancer Res.

[16] Imai M, Landen C, Ohta R, Cheung NK, Tomlinson S (2005). Complement-mediated mechanisms in anti-GD2 monoclonal antibody therapy of murine metastatic cancer.. Cancer Res.

[17] Belsches-Jablonski AP, Biscardi JS, Peavy DR, Tice DA, Romney DA (2001). Src family kinases and HER2 interactions in human breast cancer cell growth and survival.. Oncogene.

[18] Valabrega G, Montemurro F, Aglietta M (2007). Trastuzumab: mechanism of action, resistance and future perspectives in HER2-overexpressing breast cancer.. Ann Oncol.

[19] Huang X, Gao L, Wang S, McManaman JL, Thor AD (2010). Heterotrimerization of the growth factor receptors erbB2, erbB3, and insulin-like growth factor-i receptor in breast cancer cells resistant to herceptin.. Cancer Res.

[20] Piccart-Gebhart MJ, Procter M, Leyland-Jones B, Goldhirsch A, Untch M (2005). Trastuzumab after adjuvant chemotherapy in HER2-positive breast cancer.. N Engl J Med.

[21] Green DR (1998). Apoptotic pathways: the roads to ruin.. Cell.

[22] Green DR, Reed JC (1998). Mitochondria and apoptosis.. Science.

[23] Witzig TE, Flinn IW, Gordon LI, Emmanouilides C, Czuczman MS (2002). Treatment with ibritumomab tiuxetan radioimmunotherapy in patients with rituximab-refractory follicular non-Hodgkin's lymphoma.. J Clin Oncol.

[24] Galluzzi L, Kroemer G (2008). Necroptosis: a specialized pathway of programmed necrosis.. Cell.

[25] Okada N, Yin S, Asai S, Kimbara N, Dohi N (2005). Human IgM monoclonal antibodies reactive with HIV-1-infected cells generated using a trans-chromosome mouse.. Microbiol Immunol.

[26] Ishigami T, Kim KM, Horiguchi Y, Higaki Y, Hata D (1992). Anti-IgM antibody-induced cell death in a human B lymphoma cell line, B104, represents a novel programmed cell death.. J Immunol.

[27] Nahta R, Esteva FJ (2006). HER2 therapy: molecular mechanisms of trastuzumab resistance.. Breast Cancer Res.

[28] Kumar R (2007). ErbB-dependent signaling as a determinant of trastuzumab resistance.. Clin Cancer Res.

[29] Ritter CA, Perez-Torres M, Rinehart C, Guix M, Dugger T (2007). Human breast cancer cells selected for resistance to trastuzumab in vivo overexpress epidermal growth factor receptor and ErbB ligands and remain dependent on the ErbB receptor network.. Clin Cancer Res.

[30] Anido J, Scaltriti M, Bech Serra JJ, Santiago Josefat B, Todo FR (2006). Biosynthesis of tumorigenic HER2 C-terminal fragments by alternative initiation of translation.. EMBO J.

[31] Scaltriti M, Rojo F, Ocana A, Anido J, Guzman M (2007). Expression of p95HER2, a truncated form of the HER2 receptor, and response to anti-HER2 therapies in breast cancer.. J Natl Cancer Inst.

[32] Berns K, Horlings HM, Hennessy BT, Madiredjo M, Hijmans EM (2007). A functional genetic approach identifies the PI3K pathway as a major determinant of trastuzumab resistance in breast cancer.. Cancer Cell.

[33] Pandolfi PP (2004). Breast cancer–loss of PTEN predicts resistance to treatment.. N Engl J Med.

[34] Larson RA, Sievers EL, Stadtmauer EA, Lowenberg B, Estey EH (2005). Final report of the efficacy and safety of gemtuzumab ozogamicin (Mylotarg) in patients with CD33-positive acute myeloid leukemia in first recurrence.. Cancer.

[35] Amadori S, Suciu S, Stasi R, Willemze R, Mandelli F (2005). Gemtuzumab ozogamicin (Mylotarg) as single-agent treatment for frail patients 61 years of age and older with acute myeloid leukemia: final results of AML-15B, a phase 2 study of the European Organisation for Research and Treatment of Cancer and Gruppo Italiano Malattie Ematologiche dell'Adulto Leukemia Groups.. Leukemia.

[36] Ghetie MA, Bright H, Vitetta ES (2001). Homodimers but not monomers of Rituxan (chimeric anti-CD20) induce apoptosis in human B-lymphoma cells and synergize with a chemotherapeutic agent and an immunotoxin.. Blood.

[37] Wolff EA, Schreiber GJ, Cosand WL, Raff HV (1993). Monoclonal antibody homodimers: enhanced antitumor activity in nude mice.. Cancer Res.

[38] Hughes DP, Thomas DG, Giordano TJ, Baker LH, McDonagh KT (2004). Cell surface expression of epidermal growth factor receptor and Her-2 with nuclear expression of Her-4 in primary osteosarcoma.. Cancer Res.

